# Supplementary material for: Discovery and application of insertion-deletion (INDEL) polymorphisms for QTL mapping of early life-history traits in Atlantic salmon
Source: BMC Genomics. 2010 Mar 8;11:156. doi: 10.1186/1471-2164-11-156 (PMC2838853; doi:10.1186/1471-2164-11-156)
Supplement: Additional file 2 — Information on developed 76 locus single-run INDEL panel in Atlantic salmon. Information on fluorescence labeling, primer concentrations, PCR pooling and links to alignments, INDEL motifs and GENESCAN (Burge and Karlin 1997) predictions of genes/exons are available in html format. [file 1471-2164-11-156-S2.ZIP › Additionalfile2/table3772.html]

Contig Alignment

```
Base	A B C D E F G H I J K L M N O P   
1	A . . . . . . . . . . . . . . .    
2	A . . . . . . . . . . . . . . .    
3	A . . . . . . . . . . . . . . .    
4	C . . . . . . . . . . . . . . .    
5	A . . . . . . . . . . . . . . .    
6	A . . . . . . . . . . . . . . .    
7	A . . . . . . . . . . . . . . .    
8	A . . . . . . . . . . . . . . .    
9	A . . . . . . . . . . . . . . .    
10	A . . . . . . . . . . . . . . .    
11	C . . . . . . . . . . . . . . .    
12	A . . . . . . . . . . . . . . .    
13	A . . . . . . . . . . . . . . .    
14	C . . . . . . . . . . . . . . .    
15	T . . . . . . . . . . . . . . .    
16	G . . . . . . . . . . . . . . .    
17	T . . . . . . . . . . . . . . .    
18	G . . . . . . . . . . . . . . .    
19	G . . . . . . . . . . . . . . .    
20	G . . . . . . . . . . . . . . .    
21	T . . . . . . . . . . . . . . .    
22	G . . . . . . . . . . . . . . .    
23	T . . . . . . . . . . . . . . .    
24	C . . . . . . . . . . . . . . .    
25	T . . . . . . . . . . . . . . .    
26	C . . . . . . . . . . . . . . .    
27	C . . . . . . . . . . . . . . .    
28	C . . . . . . . . . . . . . . .    
29	A . . . . . . . . . . . . . . .    
30	T . . . . . . . . . . . . . . .    
31	C . . . . . . . . . . . . . . .    
32	A . . . . . . . . . . . . . . .    
33	A . . . . . . . . . . . . . . .    
34	A . . . . . . . . . . . . . . .    
35	G . . . . . . . . . . . . . . .    
36	A . . . . . . . . . . . . . . .    
37	C . C . . . . . . . . . . . . .    
38	T . T . . . . . . . . . . . . .    
39	T . T . . . . . . . . . . . . .    
40	T . T . . . . . . . . . . . . .    
41	C . C . . . . . . . . . . . . .    
42	T . T . . . . . . . . . . . . .    
43	T . T . . . . . . . . . . . . .    
44	G . G . . . . . . . . . . . . .    
45	C . C C . . . . . . . . . . . .    
46	G . G G . . . . . . . . . . . .    
47	A . A A . . . . . . . . . A . .    
48	G . G G . . . . . . . . . G . .    
49	C . C C . . . . . . . . . C . .    
50	C . C C . . . . . . . . . C . .    
51	A . A A . . . . . . . . . A . .    
52	G . G G . . . . . . . . . G . .    
53	T . T T T . . . . . . . . T . .    
54	A . A A A . . . . . . . . A . .    
55	G . G G G . . . . . . . . G . .    
56	C . C C C . . . . . . . . C . .    
57	A . A A A . . . . . . . . A . .    
58	T . T T T . . . . . . . . T . .    
59	A . A A A . . . . . . . . A . .    
60	A . A A A . . . . . . . . A . .    
61	T . T T T . . . . . . . . T . .    
62	G . G G G . . . . . . . . G . .    
63	T . T T T . . . . . . . . T . .    
64	T . T T T . . . . . . . . T . .    
65	C . C C C . . . . . . . . C . .    
66	A . A A A A . . . . . . . A . .    
67	A . A A A A . . . . . . . A . .    
68	A . A A A A . . . . . . . A . .    
69	A . A A A A . . . . . . . A . .    
70	T . T T T T . . . . . . . T . .    
71	A . A A A A . . . . . . . A . .    
72	A A A A A A . . . . . . . A . .    
73	A A A A A A . . . . . . . A . .    
74	T T T T T T . . . . . . . T . .    
75	G G G G G G . . . . . . . G . .    
76	A A A A A A . . . . . . . A . .    
77	T T T T T T . . . . . . . T . .    
78	A A A A A A . . . . . . . A . .    
79	A A A A A A . . . . . . . A . .    
80	T T T T T T . . . . . . . T . .    
81	C C C C C C . . . . . . . C . .    
82	T T T T T T . . . . . . . T . .    
83	G G G G G G . . . . . . . G . .    
84	A A A A A A . . . . . . . A . .    
85	T T T T T T . . . . . . . T . .    
86	T T T T T T . . . . . . . T . .    
87	A A A A A A . . . . . . . A . .    
88	T T T T T T . . . . . . . T . .    
89	G G G G G G . . . . . . . G . .    
90	G G G G G G . . . . . . . G G .    
91	A A A A A A . . . . . . . A A .    
92	T T T T T T . . . . . . . T T .    
93	C C C C C C . . . . . . . C C .    
94	A A A A A A . . . . . . . A A .    
95	T T T T T T . . . . . . . T T .    
96	C C C C C C . . . . . . . C C .    
97	A A A A A A . . . . . . . A A .    
98	T T T T T T . . . . . . . T T .    
99	C C C C C C . . . . . . . C C .    
100	T T T T T T . . . . . . . T T .    
101	G G G G G G . . . . . . . G G .    
102	G G G G G G . . . . . . . G G .    
103	A A A A A A . . . . . . . A A .    
104	T T T T T T . . . . . . . T T .    
105	C C C C C C . . . . . . . C C .    
106	T T T T T T . . . . . . . T T .    
107	A A A A A A . A . . . . . A A .    
108	T T T T T T . T . . . . . T T .    
109	T T T T T T . T . . T . . T T .    
110	C C C C C C . C . . C . . C C .    
111	T T T T T T . T . . T . . T T .    
112	G G G G G G . G . . G . . G G .    
113	A A A A A A . A . . A . . A A .    
114	C C C C C C . C . . C . . C C .    
115	C C C C C C . C . . C . . C C .    
116	A A A A A A . A . . A . . A A .    
117	A A A A A A . A . . A . . A A .    
118	T T T T T T . T . . T . . T T .    
119	G G G G G G . G . . G . . G G .    
120	T T T T T T . T . . T . . T T .    
121	A A A A A A . A . . A . . A A .    
122	C C C C C C . C . . C . . C C .    
123	C C C C C C . C . C C . . C C .    
124	T T T T T T . T . T T . . T T .    
125	T T T T T T . T . T T . . T T .    
126	A A A A A A . A . A A . . A A .    
127	T T T T T T . T . T T . . T T .    
128	T T T T T T . T . T T . . T T .    
129	T T T T T T . T . T T . . T T .    
130	G G G G G G . G . G G . . G G .    
131	A A A A A A . A . A A . . A A .    
132	C C C C C C . C . C C . . C C .    
133	T T T T T T . T . T T . . T T .    
134	C C C C C C . C . C C . . C C .    
135	G G G G G G . G . G G . . G G .    
136	A A A A A A . A . A A . . A A .    
137	A A A A A A . A . A A . . A A .    
138	T T T T T T . T . T T . . T T .    
139	A A A A A A . A . A A . . A A .    
140	T T T T T T . T . T T . . T T .    
141	A A A A A A . A . A A . . A A .    
142	C C C C C C . C . C C . . C C .    
143	A A A A A A . A . A A . . A A .    
144	A A A A A A . A . A A . . A A .    
145	T T T T T T . T . T T . . T T .    
146	C C C C C C . C . C C . . C C .    
147	A A A A A A . A . A A . . A A .    
148	A A A A A A . A . A A . . A A .    
149	G G G G G G . G . G G . . G G .    
150	T T T T T T . T . T T . . T T .    
151	A A A A A A . A . A A . . A A .    
152	C C C C C C . C . C C . . C C .    
153	A A A A A A . A . A A . . A A .    
154	A A A A A A . A . A A . . A A .    
155	C C C C C C . C . C C . . C C .    
156	G G G G G G . G . G G . . G G .    
157	G G G G G G . G . G G . . G G .    
158	A A A A A A . A . A A . . A A .    
159	C C C C C C . C . C C . . C C .    
160	A A A A A A . A . A A . . A A .    
161	C C C C C C . C . C C . . C C .    
162	T T T T T T . T . T T . . T T .    
163	C C C C C C . C . C C . . C C .    
164	C C C C C C . C . C C . . C C .    
165	C C C C C C . C . C C . . C C .    
166	T T T T T T . T . T T . . T T .    
167	G G G G G G . G . G G . . G G .    
168	T T T T T T . T . T T . . T T .    
169	G G G G G G . G . G G . . G G .    
170	A A A A A A . A . A A . . A A .    
171	T T T T T T T T . T T . . T T .    
172	T T T T T T T T . T T . . T T .    
173	T T T T T T T T . T T . . T T .    
174	A A A A A A A A . A A . . A A .    
175	T T T T T T T T . T T . . T T .    
176	A A A A A A A A . A A . . A A .    
177	A A A A A A A A . A A . . A A .    
178	T T T T T T T T . T T . . T T .    
179	T T T T T T T T . T T . . T T .    
180	T T T T T T T T . T T . . T T .    
181	A A A A A A A A . A A . . A A .    
182	C C C C C C C C . C C . . C C .    
183	T T T T T T T T . T T . . T T .    
184	G G G G G G G G . G G . . G G .    
185	A A A A A A A A . A A . . A A .    
186	A A A A A A A A . A A . . A A .    
187	G G G G G G G G . G G . . G G .    
188	A A A A A A A A . A A . . A A .    
189	A A A A A A A A . A A . . A A .    
190	A A A A A A A A . A A . . A A .    
191	T T T T T T T T . T T . . T T .    
192	A A A A A A A A . A A . . A A .    
193	C C C C C C C C . C C . . C C .    
194	G G G G G G G G . G G . . G G .    
195	T T T T T T T T . T T . . T T .    
196	T T T T T T T T . T T . . T T .    
197	T T T T T T T T . T T . . T T .    
198	G G G G G G G G . G G . . G G .    
199	T T T T T T T T . T T . . T T .    
200	C C C C C C C C . C C . . C C .    
201	A A A A A A A A . A A . . A A .    
202	A A A A A A A A . A A . . A A .    
203	A A A A A A A A . A A . . A A .    
204	T T T T T T T T . T T . . T T .    
205	A A A A A A A A . A A . . A A .    
206	T T T T T T T T . T T . . T T .    
207	T T T T T T T T . T T . . T T .    
208	C C C C C C C C . C C . . C C .    
209	A A A A A A A A . A A . . A A .    
210	A A A A A A A A . A A . . A A .    
211	G G G G G G G G . G G . . G G .    
212	T T T T T T T T . T T . . T T .    
213	T T T T T T T T . T T . . T T .    
214	T T T T T T T T . T T . . T T .    
215	C C C C C C C C . C C . . C C .    
216	T T T T T T T T . T T . . T T .    
217	G G G G G G G G . G G . . G G .    
218	C C C C C C C C . C C . . C C .    
219	C C C C C C C C C C C . . C C .    
220	A A A A A A A A A A A . A A A .    
221	G G G G G G G G G G G G G G G .    
222	A A A A A A A A A A A A A A A .    
223	A A A A A A A A A A A A A A A .    
224	A A A A A A A A A A A A A A A .    
225	T T T T T T T T T T T T T T T .    
226	G G G G G G G G G G G G G G G .    
227	A A A A A A A A A A A A A A A .    
228	T T T T T T T T T T T T T T T .    
229	G G G G G G G G G G G G G G G .    
230	C C C C C C C C C C C C C C C .    
231	T T T T T T T T T T T T T T T .    
232	G G G G G G G G G G G G G G G .    
233	T T T T T T T T T T T T T T T .    
234	A A A A A A A A A A A A A A A .    
235	G G G G G G G G G G G G G G G .    
236	T T T T T T T T T T T T T T T .    
237	C C C C C C C C C C C C C C C .    
238	T T T T T T T T T T T T T T T .    
239	T T T T T C T T T T T T T T T .    
240	T T T T T T T T T T T T T T T .    
241	G G G G G G G G G G G G G G G .    
242	A A A A A A A A A A A A A A A .    
243	G G G G G G G G G G G G G G G .    
244	G G G G G G G G G G G G G G G .    
245	A A A A A A A A A A A A A A A .    
246	T T T T T T T T T T T T T T T .    
247	C C C C C C C C C C C C C C C .    
248	T T T T T T T T T T T T T T T .    
249	T T T T T T T T T T T T T T T .    
250	G G G G G G G G G G G G G G G .    
251	T T T T T T T T T T T T T T T .    
252	T T T T T T T T T T T T T T T .    
253	C C C C C C C C C C C C C C C .    
254	C C C C C C C C C C C C C C C .    
255	A A A A A A A A A A A A A A A .    
256	A A A A A A A A A A A A A A A .    
257	G G G G G G G G G G G G G G G .    
258	T T T T T T T T T T T T T T T .    
259	A A A A A A A A A A A A A A A .    
260	G G G G G G G G G G G G G G G .    
261	C C C C C C C C C C C C C C C .    
262	C C C C C C C C C C C C C C C .    
263	T T T T T T T T T T T T T T T .    
264	A A A A A A A A A A A A A A A .    
265	C C C C C C C C C C C C C C C .    
266	T T T T T T T T T T T T T T T .    
267	T T T T T T T T T T T T T T T .    
268	G G G G G G G G G G G G G G G .    
269	A A A A A A A A A A A A A A A .    
270	G G G G G G G G G G G G G G G .    
271	T T T T T T T T T T T T T T T .    
272	G G G G G G G G G G G G G G G .    
273	T T T T T T T T T T T T T T T .    
274	C C C C C C C C C C C C C C C .    
275	G G G G G G G G G G G G G G G .    
276	G G G G G G G G G G G G G G G .    
277	G G G G G G G G G G G G G G G .    
278	G G G G G G G G G G G G G G G .    
279	G G G G G G G G G G G G G G G .    
280	C C C C C C C C C C C C C C C .    
281	C C C C C C C C C C C C C C C .    
282	A A A A A A A A A A A A A A A .    
283	A A A A A A A A A A A A A A A .    
284	G G G G G G G G G G G G G G G .    
285	G G G G G G G G G G G G G G G .    
286	C C C C C C C C C C C C C C C .    
287	T T T T T T T T T T T T T T T .    
288	T T T T T T T T T T T T T T T .    
289	G G G G G G G G G G G G G G G .    
290	T T T T T T T T T T T T T T T .    
291	T T T G T T T T T T T T T T T .    
292	G G G G G G G G G G G G G G G .    
293	A A A A A A A A A A A A A A A .    
294	T T T T T T T T T T T T T T T .    
295	A A A A A A A A A A A A A A A .    
296	A A A A A A A A A A A A A A A .    
297	G G G G G G G G G G G G G G G .    
298	C C C C C C C C C C C C C C C .    
299	A A A A A A A A A A A A A A A .    
300	A A A A A A A A A A A A A A A .    
301	C C C C C C C C C C C C C C C .    
302	C C C C C C C C C C C C C C C .    
303	A A A A A A A A A A A A A A A .    
304	G G G G G G G G G G G G G G G .    
305	T T T T T T T T T T T T T T T .    
306	G G G G G G G G G G G G G G G .    
307	T T T T T T T T T T T T T T T .    
308	G G G G G G G G G G G G G G G .    
309	T T T T T T T T T T T T T T T .    
310	T T T T T T T T T T T T T T T .    
311	T T T T T T T T T T T T T T T .    
312	G G G G G G G G G G G G G G G .    
313	G G G G G G G G G G G G G G G .    
314	C C C C C C C C C C C C C C C .    
315	A A A A A A A A A A A A A A A .    
316	A A A A A A A A A A A A A A A .    
317	T T T T T T T T T T T T T T T .    
318	T T T T T T T T T T T T T T T .    
319	T T T T T T T T T T T T T T T .    
320	A A A A A A A A A A A A A A A .    
321	T T T T T T T T T T T T T T T .    
322	G G G G G G G G G G G G G G G .    
323	G G G G G G G G G G G G G G G .    
324	A A A A A A A A A A A A A A A .    
325	A A A A A A A A A A A A A A A .    
326	T T T T T T T T T T T T T T T .    
327	C C C C C C C C C C C C C C C .    
328	T T T T T T T T T T T T T T T .    
329	G G G G G G G G G G G G G G G .    
330	T T T T T T T T T T T T T T T .    
331	G G G G G G G G G G G G G G G .    
332	G G G G G G G G G G G G G G G .    
333	A A A A A A A A A A A A A A A .    
334	G G G G G G G G G G G G G G G .    
335	G G G G G G G G G G G G G G G .    
336	G G G G G G G G G G G G G G G .    
337	A A A A A A A A A A A A A A A .    
338	A A A A A A A A A A A A A A A .    
339	A A A A A A A A A A A A A A A .    
340	T T T T T T T T T T T T T T T .    
341	G G G G G G G G G G G G G G G G    
342	T T T T T T T T T T T T T T T T    
343	A A A G A G A A A A A A A A A A    
344	G G G G G G G G G G G G G G G G    
345	C C C C C C C C C C C C C C C C    
346	C C C C C C C C C C C C C C C C    
347	C C C C C C C C C C C C C C C C    
348	T T T T T T T T T T T T T T T T    
349	A A A A A A A A A A A A A A A A    
350	T T T T T T T T T T T T T T T T    
351	A A A A A A A A A A A A A A A A    
352	G G G G G G G G G G G G G G G G    
353	C C C C C C C C C C C C C C C C    
354	A A A A A A A A A A A A A A A A    
355	G G G G G G G G G G G G G G G G    
356	A A A A A A A A A A A A A A A A    
357	A A A A A A A A A A A A A A A A    
358	A A A A A A A A A A A A A A A A    
359	G G G G G G G G G G G G G G G G    
360	C C C C C C C C C C C C C C C C    
361	A A A A A A A A A A A A A A A A    
362	G G G G G G G G G G G G G G G G    
363	T T T T T T T T T T T T T T T T    
364	G G G G G G G G G G G G G G G G    
365	C C C C C C C C C C C C C C C C    
366	T T T T T T T T T T T T T T T T    
367	C C C C C C C C C C C C C C C C    
368	A A A A A A A A A A A A A A A A    
369	A A A A A A A A A A A A A A A A    
370	A A A A A A A A A A A A A A A A    
371	A A - A A A A A A A A A A A A A    
372	G G G G G G G G G G G G G G G G    
373	G G G G G G G G G G G G G G G G    
374	G G G G G G G G G G G G G G G G    
375	A A A A A A A A A A A A A A A A    
376	A A A A A A A A A A A A A A A A    
377	A A A A A A A A A A A A A A A A    
378	A A A A A A A A A A A A A A A A    
379	G G G G G G G G G G G G G G G G    
380	C C C C C C C C C C C C C C C C    
381	A A A A A A A A A A A A A A A A    
382	A A A A A A A A A A A A A A A A    
383	C C C C C C C C C C C C C C C C    
384	G G G G G G G G G G G G G G G G    
385	T T T T T T T T T T T T T T T T    
386	A A A A A A A A A A A A A A A A    
387	C C C C C C C C C C C C C C C C    
388	A A A A C A A A A A A A A A A A    
389	A A A A A A A A A A A A A A A A    
390	C C C C C C C C C C C C C C C C    
391	C C C C C C C C C C C C C C C C    
392	T T T T T T T T T T T T T T T T    
393	C C C C C C C C C C C C C C C C    
394	G G G G G G G G G G G G G G G G    
395	A A A A A A A A A A A A A A A A    
396	G G G G G G G G G G G G G G G G    
397	T T T T T T T T T T T T T T T T    
398	T T T T T T T T T T T T T T T T    
399	T T T T T T T T T T T T T T T T    
400	T T T T T T T T T T T T T T T T    
401	A A A A A A A A A A A A A A A A    
402	G G G G G G G G G G G G G G G G    
403	T T T T T T T T T T T T T T T T    
404	G G G G G G G G G G G G G G G G    
405	A A A A A A A A A A A A A A A A    
406	A A A A A A A A A A A A A A A A    
407	G G G G G G G G G G G G G G G G    
408	C C C C C C C C C C C C C C C C    
409	T T T T T T T T T T T T T T T T    
410	T T T T T T T T T T T T T T T T    
411	T T T T T T T T T T T T T T T T    
412	A A A A A A A A A A A A A A A A    
413	T T T T T T T T T T T T T T T T    
414	C C C C C C C C C C C C C C C C    
415	C C C C C C C C C C C C C C C C    
416	C C C C C C C C C C C C C C C C    
417	C C C C C C C C C C C C C C C C    
418	T T T T T T T T T T T T T T T T    
419	T T T T T T T T T T T T T T T T    
420	A A A A A A A A A A A A A A A A    
421	T T T T T T T T T T T T T T T T    
422	A A A A A A A A A A A A A A A A    
423	T T T T T T T T T T T T T T T T    
424	G G G G G G G G G G G G G G G G    
425	G G G G G G G G G G G G G G G G    
426	T T T T T T T T T T T T T T T T    
427	C C C C C C C C C C C C C C C C    
428	A A A A A A A A A A A A A A A A    
429	C C C C C C C C C C C C C C C C    
430	A A A A A A A A A A A A A A A A    
431	T T T T T T T T T T T T T T T T    
432	T T T T T T T T T T T T T T T T    
433	G G G G G G G G G G G G G G G G    
434	C C C C C C C C C C C C C C C C    
435	A A A A A A A A A A A A A A A A    
436	T T T T T T T T T T T T T T T T    
437	T T T T T T T T T T T T T T T T    
438	A A A A A A A A A A A A A A A A    
439	T T T T T T T T T T T T T T T T    
440	T T T T T T T T T T T T T T T T    
441	A A A A A A A A A A A A A A A A    
442	A A A A A A A A A A A A A A A A    
443	T T T T T T T T T T T T T T T T    
444	G G G G G G G G G G G G G G G G    
445	T T T T T T T T T T T T T T T T    
446	G G G G G G G G G G G G G G G G    
447	A A A A A A A A A A A A A A A A    
448	A A A A A A A A A A A A A A A A    
449	A A A A A A A A A A A A A A A A    
450	G G G G G G G G G G G G G G G G    
451	A A A A A A A A A A A A A A A A    
452	G G G G G G G G G G G G G G G G    
453	G G G G G G G G G G G G G G G G    
454	T T T T T T T T T T T T T T T T    
455	T T T T T T T T T T T T T T T T    
456	A A A A A A A A A A A A A A A A    
457	T T T T T T T T T T T T T T T T    
458	T T T T T T T T T T T T T T T T    
459	G G G G G G G G G G G G G G G G    
460	C C C C C C C C C C C C C C C C    
461	C C C C C C C C C C C C C C C C    
462	C C C C C C C C C C C C C C C C    
463	A A A A A A A A A A A A A A A A    
464	A A A A A A A A A A A A A A A A    
465	C C C C C C C C C C C C C C C C    
466	T T T T T T T T T T T T T T T T    
467	T T T T T T T T T T T T T T T T    
468	T T T T T T T T T T T T T T T T    
469	C C C C C C C C C C C C C C C C    
470	A A A A A A A A A A A A A A A A    
471	A A A A A A A A A A A A A A A A    
472	G G G G G G G G G G G G G G G G    
473	C C C C C C C C C C C C C C C C    
474	C C C C C C C C C C C C C C C C    
475	A A A A A A A A A A A A A A A A    
476	A A A A A A A A A A A A A A A A    
477	G G G G G G G G G G G G G G G G    
478	A A A A A A A A A A A A A A A A    
479	T T T T T T T T T T T T T T T T    
480	T T T T T T T T T T T T T T T T    
481	C C C C C C C C C C C C C C C T    
482	T T T T T T T T T T T T T T T T    
483	A A A A A A A A A A A A A A A A    
484	C C C C C C C C C C C C C C C C    
485	T T T T T T T T T T T T T T T T    
486	T T T T T T T T T T T T T T T T    
487	G G G G G G G G G G G G G G G G    
488	C C C C C C C C C C C C C C C C    
489	T T T T T T T T T T T T T T T T    
490	C C C C C C C C C C C C C C C C    
491	A A A A A A A A A A A A A A A A    
492	A A A A A A A A A A A A A A A A    
493	A A A A A A A A A A A A A A A A    
494	G G G G G G G G G G G G G G G G    
495	T T T T T T T T T T T T T T T T    
496	G G G G G G G G G G G G G G G G    
497	T T T T T T T T T T T T T T T T    
498	T T T T T T T T T T T T T T T T    
499	G G G G G G G G G G G G G G G G    
500	G G G G G G G G G G G G G G G G    
501	A A A A A A A A A A A A A A A A    
502	G G G G G G G G G G G G G G G G    
503	C C C C C C C C C C C C C C C C    
504	C C C C C C C C C C C C C C C C    
505	T T T T T T T T T T T T T T T T    
506	A A A A A A A A A A A A A A A A    
507	C C C C C C C C C C C C C C C C    
508	T T T T T T T T T T T T T T T T    
509	T T T T T T T T T T T T T T T T    
510	G G G G G G G G G G G G G G G G    
511	A A A A A A A A A A A A A A A A    
512	C C C C C C C C C C C C C C C C    
513	T T T T T T T T T T T T T T T T    
514	G G G G G G G G G G G G G G G G    
515	T T T T T T T T T T T T T T T T    
516	T T T T T T T T T T T T T T T T    
517	T T T T T T T T T T T T T T T T    
518	T T T T T T T T T T T T T T T T    
519	G G G G G G G G G G G G G G G G    
520	A A A A A A A A A A A A A A A A    
521	A A A A A A A A A A A A A A A A    
522	G G G G G G G G G G G G G G G G    
523	A A A A A A A A A A A A A A A A    
524	G G G G G G G G G G G G G G G G    
525	T T T T T T T T T T T T T T T T    
526	A A A A A A A A A A A A A A A A    
527	A A A A A A A A A A A A A A A A    
528	G G G G G G G G G G G G G G G G    
529	C C C C C C C C C C C C C C C C    
530	C C C C C C C C C C C C C C C C    
531	T T T T T T T T T T T T T T T T    
532	T T T T T T T T T T T T T T T T    
533	A A A A A A A A A A A A A A A A    
534	G G G G G G G G G G G G G G G G    
535	A A A A A A A A A A A A A A A A    
536	C C C C C C C C C C C C C C C C    
537	T T T T T T T T T T T T T T T T    
538	A A A A A A A A A A A A A A A A    
539	C C C C C C C C C C C C C C C C    
540	A A A A A A A A A A A A A A A A    
541	T T T T T T T T T T T T T T T T    
542	T T T C T T T T T T T - T T T T    
543	T T T T T T T T T T T T T T T T    
544	T T T T T T T T T T T T T T T T    
545	T T T T T T T T T T T T T T T T    
546	T T T T T T T T T T T T T T T T    
547	T T T T T T T T T T T T T T T T    
548	T T T T T T T T T T T T T T T T    
549	G G G G G G G G G G G G G G G G    
550	G G G G G G G G G G G G G G G G    
551	C C C C C C C C C C C C C C C C    
552	C C C C C C C C C C C C C C C C    
553	A A A A A A A A A A A A A A A A    
554	A A A A A A A A A A A A A A A A    
555	G G G G G G G G G G G G G G G G    
556	T T T T T C T T T T T T T T T T    
557	T T T T T T T T T T T T T T T T    
558	G G G G G G G G G G G G G G G G    
559	C C C C C C C C C C C C C C C C    
560	T T T T T T T T T T T T T T T T    
561	C C C C C C C C C C C C C C C T    
562	T T T T T T T T T T T T T T T T    
563	C C C C C C C C C C C C C C C C    
564	A A A A A A A A A A A A A A A A    
565	T T T T T T T T T T T T T T T T    
566	A A A A A A A A A A A A A A A A    
567	C C C C C C C C C C C C G C C C    
568	T T T T T T T T T T T T T T T T    
569	G G G G G G G G G G G G G G G G    
570	A A A A A A A A A A A A A A A A    
571	T T T T T T T T T T T T T T T T    
572	C C C C C C C C C C C C C C C C    
573	A A A A A A A A A A A A A A A A    
574	A A A A A A A A A A A A A A A A    
575	A A A A A A A A A A A A A A A A    
576	A A A A A A A A A A A A A A A A    
577	A A A A A A A A A A A A A A A A    
578	G G G G G G G G G G G G G G G G    
579	G G G G G G G G G G G G G G G G    
580	C C C C C C C C C C C C C C C C    
581	T T T T T T T T T T T T T T T T    
582	G G G C G G G G G G G G G G G G    
583	T T T T T T T T T T T T T T T T    
584	C C C C C C C C C C C C C C C C    
585	C C C C C C C C C C C C C C C C    
586	A A A A A A A A A A A A A A A A    
587	T T T T T T T T T T T T T T T T    
588	C C C C C C C C C C C C C C C C    
589	C C C C C C C C C C C C C C C C    
590	T T T T T T T T T T T T T T T T    
591	G G G G G G G G G G G G G G G G    
592	C C C C C C C C C C C C C C C C    
593	C C C C C C C C C C C C C C C C    
594	T T T T T T T T T T T T T T T T    
595	C C C C C C C C C C C C C C C C    
596	C C C C C C C C C C C C C C C C    
597	A A A A A A A A A A A A A A A A    
598	G G G G G G G G G G G G G G G G    
599	T T T T T T T T T T T T T T T T    
600	G G G G G G G G G G G G G G G G    
601	G G G G G G G G G G G G G G G G    
602	G G G G G G G G G G G G G G G G    
603	T T T T T T T T T T T T T T T T    
604	T T T T T T T T T T T T T T T T    
605	T T T T T T T T T T T T T T T T    
606	T T T T T T T T T T T T T T T T    
607	T T T T T T T T T T T T T T T T    
608	T T T T T T T T T T T T T T T T    
609	T T T T T T T T T T T T T T T T    
610	T - - - - - - - - - - - - - - -    
611	G G G G G G G G G G G G G G G G    
612	T T T T T T T T T T T T T T T T    
613	G G G G G G G G G G G G G G G G    
614	T T T T T T T T T T T T T T T T    
615	G G G G G G G G G G G G G G G G    
616	T T T T T T T T T T T T T T T T    
617	A A A A A A A A A A A A A A A A    
618	A A A A A A A A A A A A A A A A    
619	A A A A A A A A A A A A A A A A    
620	T T T T T T T T T T T T T T T T    
621	T T T T T T T T T T T T T T T T    
622	G G G G G G G G G G G G G G G G    
623	T T T T T T T T T T T T T T T T    
624	T T T T T T T T T T T T T T T T    
625	G G G G G G G G G G G G G G G G    
626	A A A A A A A A A A A A A A A A    
627	C C C C C C C C C C C C C C C C    
628	A A A A A A A A A A A A A A A A    
629	T T T T T T T T T T T T T T T T    
630	T T T T T T T T T T T T T T T T    
631	G G G G G G G G G G G G G G G G    
632	C C C C C C C C C C C C C C C C    
633	A A A A A A A A A A A A A A A A    
634	T T T T T T T T T T T T T T T T    
635	T T T T T T T T T T T T T T T T    
636	A A A A A A A A A A A A A A A A    
637	A A A A A A A A A A A A A A A A    
638	G G G G G G G G G G G G G G G G    
639	A A A A A A A A A A A A A A A A    
640	T T T T T T T T T T T T T T T T    
641	G G G G G G G G G G G G G G G G    
642	A A A A A A A A A A A A A A A A    
643	C C C C C C C C C C C C C C C C    
644	A A A G A A A A A A A A A A A A    
645	G G G G G G G G G G G G G G G G    
646	G G G G G G G G G G G G G G G G    
647	G G G G G G G G G G G G G G G G    
648	T T T T T T T T T T T T T T T T    
649	C C C C C C C C C C C C C C C T    
650	T T T T T T T T T T T T T T T T    
651	G G G G G G G G G G G G G G G G    
652	C C C C C C C C C C C C C C C C    
653	A A A A A A A A A A A A A A A A    
654	G G G G G G G G G G G G G G G G    
655	C C C C C C C C C C C C C C C C    
656	A A A A A A A A A A A A A A A A    
657	C C C C C C C C C C C C C C C C    
658	T T T T T T T T T T T T T T T T    
659	G G G G G G G G G G G G G G G G    
660	T T T T T T T T T T T T T T T T    
661	- G - - - - G - - - - - - G G G  5	 indel  
662	- T - - - - T - - - - - - T T T  5	 indel  
663	A A A A A A A A A A A A A A A A    
664	A A A A A A A A A A A A A A A A    
665	A A A A A A A A A A A A A A A A    
666	G G G G G G G G G G G G G G G G    
667	A A A A A A A A A A A A A A A A    
668	A A A A A A A A A A A A A A A A    
669	T T T T T T T T T T T T T T T T    
670	G G G G G G G G G G G G G G G G    
671	A A A A A A A A A A A A A A A A    
672	A A A A A A A A A A A A A A A A    
673	T T T T T T T T T T T T T T T T    
674	G G G G G G G G G G G G G G G G    
675	C C C C C C C C C C C C C C C C    
676	A A A A A A A A A A A A A A A A    
677	T T T T T T T T T T T T T T T T    
678	T T T T T T T T T T T T T T T T    
679	T T T T T T T T T T T T T T T T    
680	A A A A A A A A A A A A A A A A    
681	T T T T T T T T T T T T T T T T    
682	T T T T T T T T T T T T T T T T    
683	T T T T T T T T T T T T T T T T    
684	C C C C C C C C C C C C C C C C    
685	A A A A A A A A A A A A A A A A    
686	C C C C C C C C C C C C C C C C    
687	A A A A A A A A A A A A A A A A    
688	T T T T T T T T T T T T T T T T    
689	A A A A A A A A A A A A A A A A    
690	T T T T T T T T T T T T T T T T    
691	T T T T T T T T T T T T T T T T    
692	G G G G G G G G G G G G G G G G    
693	T T T T T T T T T T T T T T T T    
694	A A A A A A A A A A A A A A A A    
695	T T T T T T T T T T T T T T T T    
696	A A A A A A A A A A A A A A A A    
697	T T T T T T T T T T T T T T T G    
698	G G G G G G G G G G G G G G G G    
699	G G G G G G G G G G G G G G G G    
700	A A A A A A A A A A A A A A A A    
701	G G G G G G G G G G G G G G G G    
702	T T T T T T T T T T T T T T T T    
703	T T T T T T T T T T T T T T T T    
704	T T T T T T T T T T T T T T T T    
705	C C C C C C C C C C C C C C C C    
706	A A A A A A A A A A A A A A A A    
707	A A A A A A A A A A A A A A A A    
708	A A A A A A A A A A A A A A A A    
709	A A A A A A A A A A A A A A A A    
710	A A A A A A A A A A A A A A A A    
711	G G G G G G G G G G G G G G G G    
712	A A A A A A A A A A A A A A A A    
713	C C C C C C C C C C C C C C C C    
714	C C C C C C C C C C C C C C C C    
715	C C C C C C C C C C C C C C C C    
716	C C C C C C C C C C C C C C C C    
717	A A A A A A A A A A A A A A A A    
718	A A A A A A A A A A A A A A A A    
719	T T T T T T T T T T T T T T T T    
720	A A A A A A A A A A A A A A A A    
721	G G G G G G G G G G G G G G G G    
722	T T T T T T T T T T T T T T T T    
723	G G G G G G G G G G G G G G G G    
724	C C C C C C C C C C C C C C C C    
725	A A A A A A A A A A A A A A A A    
726	A A A A A A A A A A A A A A A A    
727	T T T T T T T T T T T T T T T T    
728	A A A A A A A A A A A A A A A A    
729	T T T T T T T T T T T T T T T T    
730	A A A A A A A A A A A A A A A A    
731	T T T T T T T T T T T T T T T T    
732	T T T T T T T T T T T T T T T T    
733	T T T T T T T T T T T T T T T T    
734	T T T T T T T T T T T T T T T T    
735	T T T T T T T T T T T T T T T T    
736	A A A A A A A A A A A A A A A A    
737	T T T T T T T T T T T T T T T T    
738	G G G G G G G G G G G G G G G G    
739	T T T T T T T T T T T T T T T T    
740	T T T T T T T T T T T T T T T T    
741	T T T T T T T T T T T T T T T T    
742	T T T T T T T T T T T T T T T T    
743	C C C C C C C C C C C C C C C C    
744	A A A A A A A A A A A A A A A A    
745	A A A A A A A A A A A A A A A A    
746	G G G G G G G G G G G G G G G G    
747	T T T T T T T T T T T T T T T T    
748	T T T T T T T T T T T T T T T T    
749	T T T T T T T T T T T T T T T T    
750	G G G G G G G G G G G G G G G G    
751	T T T T T T T T T T T T T T T T    
752	G G G G G G G G G G G G G G G G    
753	T T T T T T T T T T T T T T T T    
754	C C C C C C C C C C C C C C C C    
755	C C C C C C C C C C C C C C C C    
756	C C C C C C C C C C C C C C C C    
757	A A A A A A A A A A A A A A A A    
758	G G G G G G G G G G G G G G G G    
759	T . T T T T T T T T T T T T T T    
760	T . T T T T T T T T T T T T T T    
761	A . A A A A A A A T A A A A A A    
762	T . T T T T T T T . T T T T T T    
763	T . T T T T T T T . T T T T T T    
764	T . T T T T T T T . T T T T T T    
765	C . C C C C C C C . C C C C C C    
766	A . A A A A A A A . A A A A A A    
767	A . A A A A A A A . A A A A A A    
768	T . T T T T T T T . T T T T T T    
769	T . T T T T G T T . T T T T T T    
770	G . G G G G . . G . G G . G G G    
771	T . T T T T . . T . T T . T T T    
772	T . T T T T . . T . T T . T T T    
773	T . T T T T . . T . T T . T T T    
774	T . T T T T . . T . T T . T T T    
775	T . T T T T . . T . T T . T T T    
776	A . A A A A . . A . A A . A A A    
777	A . A A A A . . A . A A . A A A    
778	A . A A A A . . A . A A . A A A    
779	T . T T T T . . T . T T . T T T    
780	A . A A A A . . A . A A . A A A    
781	. . . . . . . . . . . . . A A A    
782	. . . . . . . . . . . . . A A A    
783	. . . . . . . . . . . . . G G G    
784	. . . . . . . . . . . . . A A A    
785	. . . . . . . . . . . . . T T T    
786	. . . . . . . . . . . . . A A A    
787	. . . . . . . . . . . . . T T T    
788	. . . . . . . . . . . . . G G G    
789	. . . . . . . . . . . . . A A A    
790	. . . . . . . . . . . . . T T T    
791	. . . . . . . . . . . . . C C C    
792	. . . . . . . . . . . . . A A A    
793	. . . . . . . . . . . . . T T T    
794	. . . . . . . . . . . . . . A A    
795	. . . . . . . . . . . . . . A A    
796	. . . . . . . . . . . . . . T T    
797	. . . . . . . . . . . . . . G G    
798	. . . . . . . . . . . . . . T T    
799	. . . . . . . . . . . . . . A A    
800	. . . . . . . . . . . . . . T T    
801	. . . . . . . . . . . . . . A A    
802	. . . . . . . . . . . . . . G G    
803	. . . . . . . . . . . . . . C C    
804	. . . . . . . . . . . . . . C C    
805	. . . . . . . . . . . . . . . T    
806	. . . . . . . . . . . . . . . A    
807	. . . . . . . . . . . . . . . C    
808	. . . . . . . . . . . . . . . T    
809	. . . . . . . . . . . . . . . G    
810	. . . . . . . . . . . . . . . C    
811	. . . . . . . . . . . . . . . C
```
